# Colposcopic triage methods for detecting cervical intraepithelial neoplasia grade 3 after cytopathological diagnosis of low-grade squamous intraepithelial lesion: a systematic review on diagnostic tests

**DOI:** 10.1590/S1516-31802012000100008

**Published:** 2012-02-16

**Authors:** Flávia de Miranda Corrêa, Fábio Bastos Russomano, Caroline Alves de Oliveira

**Affiliations:** I MD, MSc. Senior Analyst, Cancer Control Program, Divisão de Apoio à Rede de Atenção Oncológica (DARAO), Instituto Nacional do Câncer (INCA), Rio de Janeiro, Brazil.; III MD, MSc. Gynecologist and Obstetrician, Hospital Geral de Bonsucesso, Rio de Janeiro, Brazil.; II MD, MSc, PhD. Head of Cervical Pathology Unit, Instituto Nacional de Saúde da Mulher, Criança e Adolescente Fernandes Figueira (IFF), Fundação Instituto Oswaldo Cruz (Fiocruz), Rio de Janeiro, Brazil.

**Keywords:** Cervical intraepithelial neoplasia, Triage, Colposcopy, Vaginal smears, DNA probes, HPV, Sensitivity and specificity, Neoplasia intra-epitelial cervical, Triagem.Colposcopia, Esfregaço vaginal, Sondas de DNA de HPV, Sensibilidade e especificidade.

## Abstract

**CONTEXT AND OBJECTIVE:**

The age-stratified performance of the oncogenic HPV-DNA (human papillomavirus deoxyribonucleic acid) test for triage of low-grade squamous intraepithelial lesions (LSIL) requires investigation. The objective of this study was to evaluate and compare the age-stratified performance (cutoff point: 35 years) of oncogenic HPV-DNA testing and repeated cytological tests, for detecting cervical intraepithelial neoplasia grade 3 (CIN3), in order to triage for LSIL.

**DESIGN AND SETTING:**

Systematic review. Studies were identified in nine electronic databases and in the reference lists of the articles retrieved.

**METHODS:**

The eligibility criteria consisted of initial cytological findings of LSIL; subsequent oncogenic HPV-DNA testing and repeated cytological tests; and CIN3 detection. The Quality Assessment of Diagnostic Accuracy Studies (QUADAS) guidelines were used for quality assessment. Qualitative information synthesis was performed.

**RESULTS:**

Out of 7,776 studies, 284 were identified as pertinent and three fulfilled the eligibility criteria. The CIN3 prevalence ranged from 6% to 12%. The HPV-DNA positivity rate ranged from 64% to 83%; sensitivity for CIN3 detection ranged from 95.2% to 100%; and specificity was available in two studies (27% and 52%). The sensitivity of repeated cytological tests, in relation to the threshold for atypical squamous cells of undetermined significance (ASCUS), was available in two studies (33% and 90.8%); and specificity was available in one study (53%).

**CONCLUSIONS:**

Currently, there is no scientific evidence available that would prove that colposcopic triage using oncogenic HPV-DNA testing to detect CIN3 performs better than repeated cytological tests, among women with LSIL aged 35 years and over.

## RESUMO

**CONTEXTO E OBJETIVO:**

O desempenho do teste de DNA-HPV (ácido desoxirribonucleico-papilomavírus humano) oncogênico estratificado por idade para triagem de lesão intraepitelial escamosa de baixo grau (LSIL) demanda investigação. O objetivo do estudo foi avaliar e comparar o desempenho, para detecção de neoplasia intraepitelial cervical (NIC3), do teste de DNA-HPV oncogênico e da citologia de repetição estratificados por idade (ponto de corte 35 anos), para triagem de LSIL.

**TIPO DE ESTUDO E LOCAL:**

Revisão sistemática da literatura. Foram identificados estudos em nove bases de dados eletrônicas e listas de referências dos artigos recuperados.

**MÉTODOS:**

Critérios de elegibilidade: citologia inicial LSIL, realização do teste de DNA-HPV oncogênico e da citologia de repetição e detecção de NIC3. A diretriz QUADAS (Quality Assessment of Diagnostic Accuracy Studies) foi utilizada para avaliação da qualidade. As informações foram sintetizadas qualitativamente.

**RESULTADOS:**

Dentre 7.776 estudos, 284 eram pertinentes ao tema e três atenderam aos critérios de elegibilidade. A prevalência de NIC3 apresentou variação entre 6% e 12%. A taxa de positividade do teste de DNA-HPV apresentou variação entre 64% e 83%; a sensibilidade para NIC3 apresentou variação entre 95,2% e 100%; a especificidade estava disponível em dois estudos (27% e 52%). A sensibilidade da citologia de repetição (ponto de corte ASCUS - células escamosas atípicas de significado indeterminado) estava disponível em dois estudos (33% e 90,8%); a especificidade estava disponível em um estudo (53%).

**CONCLUSÕES:**

Não existem, atualmente, evidências científicas disponíveis para comprovar que, em mulheres com 35 anos ou mais e citologia LSIL, a triagem colposcópica com teste de DNA-HPV oncogênico apresenta melhor desempenho para detecção de NIC3 do que a repetição citológica.

## INTRODUCTION

### Rationale

In 2009, according to data from the Brazilian Information System for Cervical Cancer (Sistema de Informação do Câncer do Colo do Útero, SISCOLO),^1^ low-grade squamous intraepithelial lesion (LSIL) diagnoses represented 31% of abnormal Papanicolaou (Pap) test results in Brazil. The Brazilian Ministry of Health^2^ recommends cytological test repetition six months after the initial LSIL diagnosis, with referral for colposcopy if the second cytological test shows atypical squamous cells of undetermined significance (ASC-US) or worse (ASC-US+), but there is no consensus worldwide regarding this practice.

LSIL cytology presents a challenge to guidelines or clinical recommendation outlines. The majority of these lesions spontaneously regress, thereby reflecting the cytological manifestations of human papillomavirus (HPV) infection, which are highly prevalent and transitory.^3^ However, the low sensitivity of cytological tests may cause underdiagnosis: up to 23% of LSIL cases are afterwards histologically confirmed as cervical intraepithelial neoplasia grade 2 or 3 (CIN2 or CIN3).^4^ Consequently, it is necessary to distinguish which women with LSIL cytology are at greater risk of presenting a truly potential precursor lesion. Because of the high prevalence of LSIL, colposcopic referral for all cases is not cost-effective and, above all, this may induce anxiety, overdiagnosis, overtreatment and even obstetric adverse effects.^5^ Consequently, an intermediate step between screening and colposcopy named triage has been proposed.^6^ The currently available triage methods are oncogenic HPV-DNA detection testing and cytological test repetition.

The results from the ASCUS-LSIL Triage Study (ALTS),^7^ a trial conducted by the United States National Cancer Institute, led to the conclusion that HPV-DNA testing does not serve the purpose of triage for LSIL cases well, because the positivity rate for oncogenic types in LSIL specimens is very high, thus making triage for LSIL an unproductive clinical step. These data have been confirmed by recent meta-analyses,^4^^,^^8^^,^^9^ which also demonstrated that the sensitivity of oncogenic HPV-DNA testing for CIN2 or worse (CIN2+) was not significantly higher than that of repeated cytological tests. Moreover, its specificity was substantially and statistically significantly lower.

Still, some aspects of LSIL triage deserve further consideration. The effectiveness of HPV-DNA testing for triage depends on its positivity rate, which depends on HPV infection prevalence, which in turn is age-related. In the ALTS trial, the HPV-DNA positivity rate among women with LSIL was indeed very high, but 91% of these women were less than 35 years old. Other studies^10^^,^^11^^,^^12^ found lower HPV-DNA positivity rates in women aged 30-35 years or older, and one study^12^ also concluded that triaging by means of HPV testing performed better among women aged over 35 years.

Furthermore, it is now accepted that CIN3 represents a more relevant surrogate endpoint for studies evaluating cervical cancer prevention strategies.^13^ Concerning the relative accuracy of HPV-DNA testing, compared with repeated cytological tests for LSIL triage, the endpoint was CIN2+ in the previously published meta-analyses. The points enumerated above show that efforts towards clarification are needed.

## OBJECTIVE

The main objective of the present study was to evaluate and compare the age-stratified performance (cutoff point at 35 years of age) of oncogenic HPV-DNA testing and repeated cytological tests, for histologically detecting and confirming CIN3, with the aim of undertaking colposcopic triage on women presenting initial LSIL cytological findings, through a systematic review of the literature.

## METHODS

### Protocol

This systematic review was based on a protocol developed *a priori*, which is available from the corresponding author upon request. The Preferred Reporting Items for Systematic Reviews and Meta-analyses (PRISMA) statement^14^ was used to guide the reporting of this review.

### Eligibility criteria

Studies were eligible if the women included presented initial cytological findings of LSIL, subsequently underwent oncogenic HPV-DNA testing and repeated cytological tests (independent of the technique used), and then underwent colposcopy. The endpoint was CIN3 detection and the reference standard for its verification was based on absence of abnormal colposcopic findings from satisfactory examinations or histological evaluations of tissue specimens obtained through colposcopy-directed biopsies, endocervical curettage, large loop excision of the transformation zone (LLETZ) or conization. Eligibility was not conditional on the study design, publication year or language.

### Information sources

The following electronic bibliographic databases were accessed: Medical Literature Analysis and Retrieval System Online (MEDLINE), Excerpta Medica Database (Embase), Cochrane Library (including the Cochrane Database of Systematic Reviews, Cochrane Central Register of Controlled Trials and Cochrane Groups), Web of Science, Literatura Latino Americana e do Caribe em Ciências da Saúde (LILACS), POPulation information onLINE (POPLINE), Scientific Electronic Library Online (SciELO), System for Information on Grey Literature in Europe (SIGLE) and Scopus. The searches were saved and periodically updated until the cutoff date of July 31, 2009. MEDLINE and Embase automatically retrieved newer references published after the original search. The reference lists of all the articles retrieved were also reviewed.

### Search

The search strategies were grounded in evidence-based practice guidelines^15^ and customized according to specific tools and indexed terms available in each database.

The search strategy used for the MEDLINE database was translated into the following sentence: ((“Uterine Cervical Neoplasms”[Mesh] OR “Cervical Intraepithelial Neoplasia”[Mesh] OR “Uterine Cervical Dysplasia”[Mesh]) OR (“CIN” OR “SIL” OR “LSIL” OR “Squamous Intraepithelial Lesions” OR “Low-grade Squamous Intraepithelial Lesions” OR “Low-grade Atypia” OR “Mild Atypia” OR “Mild Dyskaryosis”) OR (“Papillomaviridae”[Mesh] OR “Papillomavirus Infections”[Mesh]) OR (“HPV” OR “Human Papillomavirus”)) AND ((“DNA Probes, HPV”[Mesh] OR “Vaginal Smears”[Mesh] OR “Triage”[Mesh]) OR (“HPV-DNA” OR “Pap” OR “Papanicolaou” OR “Smear” OR “Cytology”)) AND ((“Sensitivity and Specificity”[Mesh] OR “Predictive Value of Tests”[Mesh]) OR (“Accuracy” OR “Test-positive Rate” OR “Sensitivity” OR “Specificity” OR “Positive Predictive Value” OR “Negative Predictive Value” OR “Positive Likelihood Ratio” OR “Negative Likelihood Ratio”)). The other search strategies developed can be provided upon request.

### Study selection

The first author independently screened the titles and abstracts of all the records identified, in order to evaluate their relationship to the topic. Attempts to retrieve the full text were made if the abstract was missing or did not contain sufficient information. Excluded records were assembled in separate digital files, together with the reasons for exclusion, which were documented in all cases.

Full-text articles for all potentially pertinent records were thoroughly searched. The methodology section of each study, or the full text if the necessary data were not available in the methodology section, was subsequently assessed for eligibility, in accordance with the criteria described above. A questionnaire was developed for this purpose. In the event of lack of information or uncertainty regarding inclusion or exclusion decisions, the main or corresponding author of the study was contacted by e-mail or letter, for elucidation. Excluded studies were archived together with documented reasons for exclusion, in all cases.

### Data-gathering process

Data abstraction was done using a form developed and piloted independently by the first author. Additional data were requested from the main authors by e-mail or letter when the report had insufficient data.

### Data items

The data gathered from each selected study included characteristics pertaining to the study population (study location; place, time and type of participant recruitment; inclusion and exclusion criteria; sample size; and participants’ ages), investigative tests and reference standards (technique, nomenclature, test cutoff and colposcopic referral threshold) and results (age-stratified numbers of CIN3 cases, true positives, false positives, true negatives and false negatives; and number of follow-up losses).

### Risk of bias in individual studies

The Quality Assessment of Diagnostic Accuracy Studies (QUADAS) guidelines^16^ were used for quality assessment. Two reviewers separately examined the methodology section of each study included and answered the QUADAS questionnaire, blinded to information regarding identification of authors, publication journal, funding sources, results and conclusions. Disagreements in quality assessments were resolved by means of discussion, to reach a consensus.

### Synthesis of results

A qualitative synthesis of the abstracted data was made descriptively through a structured summary on the characteristics and results from the studies included. Although envisaged in the protocol, quantitative synthesis was precluded because data for accuracy measurement calculations were available in just one study.

## RESULTS

### Study selection

**Figure 1.**
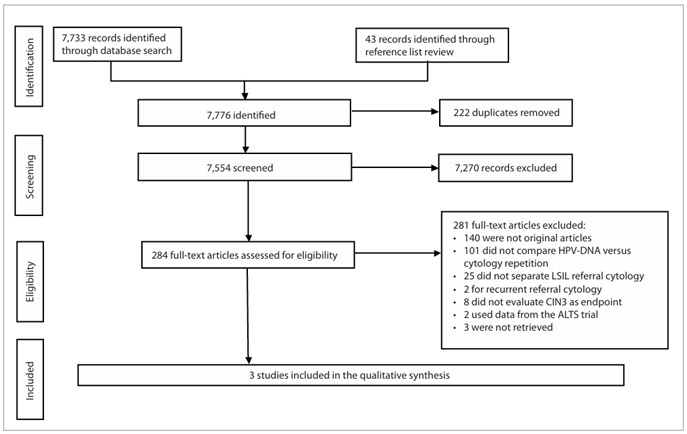
Flow diagram summarizing the results from the different phases of the systematic review.

A flow diagram detailing the process for selecting records relevant to the review is outlined in Figure 1. 

Out of the 7,776 records initially identified through the full search strategy, 222 were duplicates and were therefore removed. A total of 7,554 articles were screened and 7,270 were subsequently excluded because they were not related to the topic. The remaining 284 full-text articles were assessed for eligibility. At this point, the most common reasons for exclusion were that the studies were not original or did not compare the performance of oncogenic HPV-DNA testing and repeated cytological tests. Twenty-five studies did not separate the initial LSIL findings from other cytological diagnoses; eight studies did not evaluate the endpoint of CIN3; and three studies could not be retrieved. Consequently, these studies were excluded. Before final exclusion, the authors of 23 studies that were candidates for inclusion pending additional information or full-text retrieval were contacted. Five of these authors (22%) replied, but only one of them made available the original raw data, thereby allowing study inclusion. Three studies^7^^,^^17^^,^^18^ were included in the systematic review.

The list of all the excluded studies is available upon request.

### Study characteristics

Tables 1 and 2 summarize, respectively, the characteristics of the study populations and the investigative tests and reference standards used.

Two studies were randomized controlled trials (RCTs)^7^^,^^17^ and one had a cross-sectional design.^18^ In all the studies included, the participants were recruited consecutively within routine cervical cancer screening practice. The sample sizes ranged from 69 to 1572. There were no differences in demographic characteristics among the participants included in each arm of the RCTs (data not shown), but the cross-sectional study did not report on this subject. The participants’ mean ages ranged from 25 to 34 years. The main inclusion criterion was the initial ASCUS or LSIL cytology. The RCTs excluded women who had previously undergone cervical ablative or excisional treatment.

In all the studies included, the repeated cytological tests were classified in accordance with the 1991 Bethesda System.^19^ With regard to technique, conventional cytological tests were used in two studies^17^^,^^18^ and liquid-based cytological tests in one.^7^ The colposcopic referral threshold was ASCUS in two studies^17^^,^^18^ and high-grade squamous intraepithelial lesion (HSIL) in one.^7^

Hybrid capture-2 B-probe (HC2; Qiagen Gaithersburg, Inc., Maryland, United States; previously Digene Corporation) at a cutoff of 1 pg/ml was used in all three of the studies included, for HPV-DNA detection.

The reference standard was based on histological examination of colposcopy-directed biopsies,^7^^,^^17^^,^^18^ endocervical curettage^7^^,^^17^ and loop electrosurgical excision procedures (LEEP).^7^ Colposcopy results were accepted as negative in the absence of specimens forwarded for histological evaluation, in two studies.^7^^,^^17^ In one study,^18^ a biopsy was taken close to the squamocolumnar junction, at 12 o’clock, if there were no abnormal colposcopic findings.

### Risk of bias within studies

**Figure 2.**
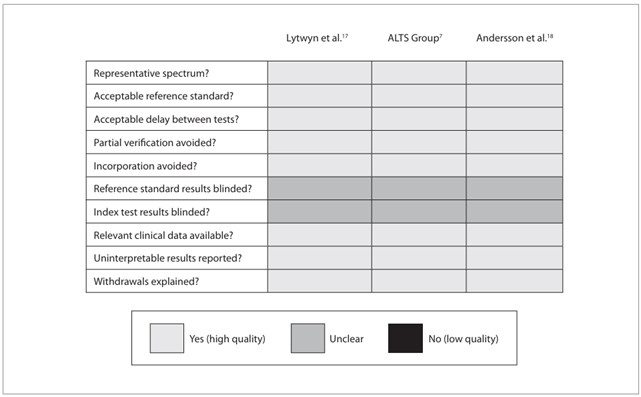
Methodological quality summary diagram. On the left side of the figure, the individual quality items are listed. On top of the figure, the individual studies included are listed. Adapted from Guide to the Graphs in a Cochrane Diagnostic Test Accuracy Review.^20^

A methodological quality diagram was used, which was adapted from the Cochrane Library Guide to the Graphs in a Cochrane Diagnostic Test Accuracy Review.^20^ This is shown in Figure 2, presenting the review authors’ judgments about the methodological quality items presented in the QUADAS questionnaire, across all the studies included.

All three studies were of high methodological quality, although the items concerning blinded interpretation of the investigative tests and reference standards used were considered unclear, from the information reported in the studies.

**Table 1.** Characteristics of the populations included in the selected studies

Authors	Lytwyn et al.,^17^ 2000^*^	ALTS Group,^7^ 2003^†^	Andersson et al.,^18^ 2005
Country	Canada	United States	Sweden
Recruitment place	52 community-based family practices and one university student health clinic in Ontario	General, gynecological or family planning clinics in Alabama, Oklahoma, Pennsylvania and Washington	Population-based screening in Stockholm
Recruitment dates	November 1995 - October 1998	January 1997 - December 1998	NI
Recruitment type	Consecutive	Consecutive	Consecutive
Inclusion criteria	- Initial cytological findings of ASCUS or LSIL - Between 16 and 50 years old	- Initial cytological findings of ASCUS or LSIL up to six before recruitment - ³ 18 years old - Capable of providing consent and probably participate throughout the duration of the study	- Initial cytological findings of ASCUS or LSIL
Exclusion criteria	- Likelihood of non-adherence to follow-up - Pregnancy - Absent cervix - Previous diagnoses of high-grade CIN , AGC-US, glandular dysplasia or cervical cancer - Previous destructive cervical treatment - Vaginal or vulvar neoplasia - Followed with colposcopy at the time of recruitment - Immunosuppression - Uterine body or adnexal surgery required	- Previous hysterectomy - Previous destructive or excisional cervical treatment - Pregnancy	NI
Sample size (at time of initial cytological finding of LSIL)	69	1572	125
Age	Average: 30 years old < 35 years old: 58 (84%) ³ 35 years old: 11 (16%)	Average: 25 years old < 35 years old: 1437 (91%) ³ 35 years old: 135 (9%)	Average: 34 years old < 35 years old: NI ³ 35 years old: NI

^*^Some data obtained from the author; ^†^Data pertaining demographic characteristics were obtained from Schiffman & Adrianza, 2000^21^; NI = Not informed; ASCUS = Atypical Squamous Cells of Undetermined Significance; LSIL = Low Grade Squamous Intraepithelial Lesions; CIN = Cervical intraepithelial neoplasia; AGC-US = Atypical Glandular Cells of Undetermined Significance.

**Table 2.** Characteristics of investigated tests and reference standard used in selected studies

Authors	Lytwyn et al.,^17^ 2000	ALTS Group,^7^ 2003	Andersson et al.,^18^ 2005
Repeated cytological test:			
Technique	Conventional	Liquid-based ThinPrep^*^	Conventional
Nomenclature	Bethesda, 1991	Bethesda, 1991	Bethesda, 1991
Colposcopic referral threshold	ASCUS, HSIL	HSIL	Any abnormality
HPV-DNA test:			
Technique	HC2	HC2	HC2
Positivity cutoff	1 pg/ml	1 pg/ml	1 pg/ml
Reference standard	- Histological examination of colposcopy-directed biopsies and/or endocervical curettage - Colposcopy taken to be negative in the absence of specimens forwarded for histological evaluation	- Histological examination of colposcopy-directed biopsies, endocervical curettage and/or LEEP - Colposcopy taken to be negative in the absence of specimens forwarded for histological evaluation	- Histological examination of colposcopy-directed biopsies (in the absence of abnormal colposcopic findings, a biopsy was taken close to the squamocolumnar junction, at 12 o’clock)

^*^Cytyc Corporation, Boxborough, Massachusetts, United States; ASCUS = Atypical Squamous Cells of Undetermined Significance; HSIL = High Grade Squamous Intraepithelial Lesions; HC2 = Hybrid Capture-2 (Qiagen Gaithersburg, Inc. Maryland, United States, previously Digene Corporation); LEEP = Loop electrosurgical excision procedure, including large loop excision of the transformation zone (LLETZ) or conization.

### Results from individual studies

Table 3 summarizes the results from the studies included. One RCT^17^ originally reported combined results for women presenting initial ASCUS/LSIL cytology, and CIN2+ as the outcome. The main author made the raw data available, which made it possible to separate out the LSIL data necessary for making accuracy measurement calculations according to age strata, CIN3 outcome and number of follow-up losses. Sixty-nine women were included: 84% were less than 35 years of age and 16% were 35 years old or over. Thirty-four were allocated to the HPV-DNA test arm and 35 to the repeated cytological test arm. Fifty women completed the study (27.5% losses); four losses (12.5%) occurred in the HPV-DNA test arm and 15 losses (43%) occurred in repeated cytological test arm. Six women presented CIN3 (12% prevalence): three in each arm of the study. All CIN3 cases were detected through a positive HPV-DNA test. The sensitivity and specificity of the HPV-DNA tests were, respectively, 100% and 52%. Regarding data availability, age-stratified HPV-DNA test performance evaluation could not be carried out, because all the women who presented CIN3 in this study arm were less than 35 years of age. The positivity rate in the HPV-DNA test was 64%. Repeated cytological tests with the ASCUS threshold detected only one of the CIN3 cases. The sensitivity and specificity of repeated cytological tests at the ASCUS threshold were, respectively, 33% and 53%.

The other RCT^7^ did not report HPV-DNA tests and repeat cytological results separately for each arm of the study. The attempts to contact the main author, to requesting data for accuracy measurement calculations did not produce any reply, and therefore only the information available in the article is reproduced here. This trial enrolled 1,572 women presenting initial cytological findings of LSIL: 91% were less than 35 years of age and 9% were 35 years of age or over. The randomization resulted in 673 women in the arm of the study with immediate colposcopic referral, 675 women in the arm with repeated cytological tests and 224 women in the arm with HPV-DNA testing. The smaller number of women placed in this last arm was because this arm was closed before the end of the study consequent to the observed high positivity rate in the HPV-DNA test (83%). Eight-two percent of the women completed the study, and the percentages of losses were similar in the three arms. The cumulative CIN3 prevalence over the two-year study period was 15%, but considering just the detection rate upon study entry, the prevalence dropped to 8%. The sensitivity for CIN3 detection defined a priori in the protocol was 55.9% in the immediate colposcopic 

**Table 3.** Numbers of true positives, true negatives, false positives and false negatives, accuracy measurements, positivity rate, age stratification and number of losses from the investigative tests; cervical intraepithelial neoplasia grade 3 (CIN3) prevalence and total losses in the selected studies

Authors	Lytwyn et al.,^17^ 2000	ALTS Group,^7^ 2003	Andersson et al.,^18^ 2005
HPV-DNA test:			
True positives	3	NA	7
False negatives	0	NA	0
False positives	13	NA	86
True negatives	14	NA	32
Sensitivity	100%	65.9%^*^	100%
Specificity	52%	NA	27%
Positivity	64%^†^	83%	74%
Age stratification	3 cases of CIN3 HPV+ < 35 years old	NA	NA
Losses	4 (12.5%)	NA	0
Repeated cytological test:			
True positives	1	NA	NA
False negatives	2	NA	NA
False positives	8	NA	NA
True negatives	9	NA	NA
Sensitivity	33%^‡^	48.4%^*^	NA
Specificity	53%^‡^	NA	NA
Losses	15 (43%)	NA	NA
CIN3 prevalence	12%^‡^	15%^§^	6%
Total losses	27.5%	18%	0

NA = Data not available; ^*^Two-year cumulative CIN3 detection; ^†^Results available for 69 patients included in the study; ^‡^Based on the number of patients who completed the study; ^§^Two-year cumulative.

referral arm, 65.9% in the HPV-DNA test arm (both taken at the time of study entry) and 48.4% in the repeated cytological test arm (including follow-up during the study, at the HSIL threshold). The HPV-DNA test sensitivity for CIN3 detection upon study entry, calculated for a theoretical situation that disregarded missing visits or tests results, was 95.2%. In the same theoretical situation, the sensitivity of the first-visit (six months after study entry) repeated cytological test for CIN3 detection was 90.8% at the ASCUS threshold. Specificities were not reported. Age-stratified HPV-DNA test accuracy evaluation could not be performed because of lack of data.

In the cross-sectional study,^18^ the results from repeated cytological tests were reported aggregated for women initially presenting cytological findings of ASCUS/LSIL, and therefore it was impossible to perform accuracy measurement calculations relative to this test in the LSIL group. There was no reply to our request for data unavailable in the published study. The results from HPV-DNA testing were reported separately for women presenting initial ASCUS and LSIL cytological findings, and accuracy measurements could be calculated. This study included 125 women presenting initial cytological findings of LSIL. These women underwent HPV-DNA testing, repeated cytological tests, colposcopy and colposcopy-directed biopsies. In cases without abnormal colposcopic findings, a biopsy was taken close to the squamocolumnar junction, at 12 o’clock. No follow-up losses occurred. The CIN3 prevalence was 6%. The HPV-DNA test was positive in the seven women who presented CIN3. No patient with a negative HPV-DNA test presented CIN3. The sensitivity and specificity of the HPV-DNA test was 100% and 27%, respectively. No evaluation of the performance of the age-stratified HPV-DNA test could be carried out because data were not available. The HPV-DNA test was positive in 93 patients (74%).

## DISCUSSION

### Summary of evidence

Following the initial cytological finding of LSIL in the studies included in the present review, the CIN3 prevalence was 6%,^18^ 8%^7^ and 12%.^17^ The rate reported in the literature is 7.4% (95% CI: 2.9-12.0%).^4^

In the studies included, the positivity rates in the oncogenic HPV-DNA tests among the women presenting initial cytological findings of LSIL were high (64%,^17^ 74%^18^ and 83%^7^), which is in agreement with the 74.4% (95% CI: 67.0-81.9%; range: 58-85%) rate reported in the literature.^4^ The HPV-DNA test sensitivities for CIN3 detection were very high (95.2%^7^ and 100%^17^^,^^18^), while the specificities were low (27%^18^ and 52%^17^). The most recent meta-analysis on this subject reported sensitivity and specificity, pooled from six studies for the same endpoint, of 97.1% (95% CI: 94.0-100%) and 26.1% (95% CI: 15.1-37.1%), respectively.^4^ Using a triage method of low specificity may induce anxiety, overdiagnosis, overtreatment and even adverse effects. As a result, more specific tests, like HPV genotyping, HPV-mRNA and p-16 have been increasingly investigated.^22^

The repeated cytological test sensitivities for CIN3 detection at the ASCUS threshold were 33%^17^ and 90.8%.^7^ The specificity was 53%.^17^ These data are not reliable because in one included study,^17^ there were selective losses in the repeat cytology arm, and in the other,^7^ they were calculated based on a theoretical situation. No reports are available in the literature to make comparisons, because in the previously published meta-analyses,^8^^,^^9^ which evaluated repeat cytological test accuracy, the endpoint was CIN2+.

Another factor that can influence triage test performance is the adherence to the proposed strategy. The potential follow-up losses need to be taken into consideration, especially if the investigation is done at a later time, as in the case of cytological test repetition. No losses occurred in the cross-sectional study,^18^ but the losses in the RCTs were 18%^7^ and 27.5%.^17^ In one study included,^17^ greater losses occurred in the repeated cytological test arm, possibly indicating that this strategy is less effective within clinical practice. On the other hand, HPV-DNA tests can be processed using the original liquid-based cytological residual specimen, thus reducing the costs and follow-up losses.

### Limitations

Data for accuracy measurement calculations were available in just one study, thus limiting the qualitative synthesis and precluding quantitative synthesis.

Even if the missing data had been obtained, age-stratified analyses would probably not have been conclusive because the studies included did not report age-specific data and were not designed to address this point. In the present review, the mean age of the women in one included study^18^ was 34 years, and only 9%^7^ and 16%^17^ of the women enrolled in the other two included studies were 35 years of age or over. As a matter of fact, although age-stratified data were available in one study,^17^ no age-stratified HPV-DNA test performance evaluation could be carried out because all the women who presented CIN3 in this arm of the study were less than 35 years old. Despite the high methodological quality attained in the QUADAS assessment, one included study^7^ failed to report the absolute numbers of false and true positives and negatives, which is considered to be a basic methodological criterion for studies relating to diagnostic test accuracy.

Further limitations occurred because, going back to the study selection step, 25 studies did not separate initial findings of LSIL from other cytological diagnoses, eight studies did not evaluate the CIN3 endpoint and three studies could not be retrieved. The attempts to contact the main or corresponding authors to request additional information or full-text retrieval achieved a low response rate indeed. Although the abstract screening on the three studies that were not retrieved suggested that they were not eligible and would not have been included, there exists the possibility of selection bias. Regarding the systematic review methodology, a very broad search was conducted in order to avoid the risk of losing potentially pertinent articles. As a result, the search achieved great sensitivity but poor specificity.

Strict observance of basic methodological criteria for designing, conducting and, particularly, reporting diagnostic test accuracy studies is essential in order to ensure quality and enable comparison between results and inclusion in systematic reviews. 

Efforts to facilitate access to relevant and valid studies, in order to develop and evaluate strategies that provide better balance between search sensitivity and accuracy, and efforts towards establishing ethical standards that may encourage the scientific community to share information are welcomed, in the pursuit of improvements in systematic review methodology.

## CONCLUSIONS

It was found that currently there is no scientific evidence available that would determine which triage method for colposcopic referral performs best in detecting CIN3 among women aged over 35 years who initially present cytological findings of LSIL. Consequently, any strategy definition will not be evidence-based. The clinical decision on which test to use in this situation should take into consideration other factors, such as patients’ values, prospects for adherence within a conservative follow-up approach, cost and technology access. The possibility of carrying out a reflex HPV-DNA test, if liquid-based cytological tests were used, may represent an advantage in clinical practice, through reducing costs and follow-up losses. Where this technology is not available or is economically impracticable, cytological test repetition still seems to be an acceptable option.

Regarding effective assessment of triage method performance in future research, uniform age-strata definition for investigation, use of the CIN3 endpoint, data separation according to initial cytological diagnoses and searching for more specific methods are the main characteristics that should be prioritized.
